# Silver nanoparticles of *Albizia adianthifolia*: the induction of apoptosis in human lung carcinoma cell line

**DOI:** 10.1186/1477-3155-11-5

**Published:** 2013-02-18

**Authors:** Rishalan Govender, Alisa Phulukdaree, Robert M Gengan, Krishnan Anand, Anil A Chuturgoon

**Affiliations:** 1 Discipline of Medical Biochemistry, School of Laboratory Medicine and Medical Sciences, University of KwaZulu-Natal, Private Bag 7, Congella, Durban, 4013, South Africa; 2 Department of Chemistry, Faculty of Applied Sciences, Durban University of Technology, Durban, 4001, South Africa

**Keywords:** Nanosilver, Albizia adianthifolia, Cancer, Apoptosis, Smac/DIABLO

## Abstract

**Background:**

Silver nanoparticles (AgNP), the most popular nano-compounds, possess unique properties. *Albizia adianthifolia* (AA) is a plant of the Fabaceae family that is rich in saponins. The biological properties of a novel AgNP, synthesized from an aqueous leaf extract of AA (AA_AgNP_), were investigated on A549 lung cells. Cell viability was determined by the MTT assay. Cellular oxidative status (lipid peroxidation and glutathione (GSH) levels), ATP concentration, caspase-3/-7, -8 and −9 activities were determined. Apoptosis, mitochondrial (mt) membrane depolarization (flow cytometry) and DNA fragmentation (comet assay) were assessed. The expression of CD95 receptors, p53, bax, PARP-1 and smac/DIABLO was evaluated by flow cytometry and/or western blotting.

**Results:**

Silver nanoparticles of AA caused a dose-dependent decrease in cell viability with a significant increase in lipid peroxidation (5-fold vs. control; *p* = 0.0098) and decreased intracellular GSH (*p* = 0.1184). A significant 2.5-fold decrease in cellular ATP was observed upon AA_AgNP_ exposure (*p* = 0.0040) with a highly significant elevation in mt depolarization (3.3-fold vs. control; *p* < 0.0001). Apoptosis was also significantly higher (1.5-fold) in AA_AgNP_ treated cells (*p* < 0.0001) with a significant decline in expression of CD95 receptors (*p* = 0.0416). Silver nanoparticles of AA caused a significant 2.5-fold reduction in caspase-8 activity (*p* = 0.0024) with contrasting increases in caspase-3/-7 (1.7-fold vs. control; *p* = 0.0180) and −9 activity (1.4-fold vs. control; *p* = 0.0117). Western blots showed increased expression of smac/DIABLO (4.1-fold) in treated cells (*p* = 0.0033). Furthermore, AA_AgNP_ significantly increased the expression of p53, bax and PARP-1 (1.2-fold; *p* = 0.0498, 1.6-fold; *p* = 0.0083 and 1.1-fold; *p* = 0.0359 respectively).

**Conclusion:**

Data suggests that AA_AgNP_ induces cell death in the A549 lung cells via the mt mediated intrinsic apoptotic program. Further investigation is required to potentiate the use of this novel compound in cancer therapy trials.

## Introduction

Cancer is a leading cause of global morbidity and mortality [1]. In 2006 there were 4525 deaths due to lung cancer in South Africa [1]. As much as 80-90% of lung cancer cases are attributed to smoking, with the smaller proportion (10-20%) as a result of occupational exposure to heavy metals [2,3]. Much recently, an association has been found between the acquired immunodeficiency syndrome (AIDS) and the development of lung cancer [4]. This is of concern considering the crisis of AIDS in South Africa. The financial strain of anti-retroviral treatment and cancer therapy necessitates the need for alternate means of cancer treatment that is cost effective, easily accessible and safe.

Nanoparticles (NPs) are small sized (1-100 nm) compounds that are able to function as whole units. These compounds are becoming widespread for their use in consumer products and medical applications; with potential for utilization as therapeutic compounds, transfection vectors, anti-microbial agents and fluorescent labels [5]. Silver NPs are the most commercialized and prominent group of nano-compounds, attributed to their diverse applications in the health sector.

Silver (Ag) possesses unique and unusual chemical, physical as well as biological properties [6]. Silver, in a colloidal form, is used for the treatment of bacterial infections in open wounds, and preparation of ointments, bandages and wound dressings [7]. Additionally, nanosilver has been used as a contraceptive, and marketed as a water disinfectant [8,9].

Silver NPs are now being exploited for the treatment of various diseases such as retinal neurovascularization [10,11] and acquired immunodeficiency syndrome as a result of human immunodeficiency virus [12]. Additionally, AgNPs are well known for their anti-microbial properties and are used as antiviral agents against hepatitis B, herpes simplex virus type 1, monkey pox virus and respiratory syncytial virus [13,14].

Concerns on environmental exposure to AgNPs have initiated toxicity studies. Silver NP-hydrogel induced DNA damage and the production of reactive oxygen species (ROS) in cultured HeLa cells [15]. A study using human lymphocytes revealed that AgNPs caused DNA damage and cell death [16]. Additionally, AgNPs induced oxidative stress and caused impairment of nuclear DNA in Swiss albino mice [16].

Recently, the use of AgNPs as anti-cancer agents has proved promising [6]. Various attempts to incorporate AgNPs into cancer treatments have been made, with positive outcomes [17]. Although the induction of oxidative stress by AgNP induced mt damage has been observed as the general mode of AgNP toxicity, mechanistic pathways remain unclear [18].

*Albizia adianthifolia*, a plant member of the Fabaceae family found abundantly on the east coast of South Africa, contains saponins such as prosapogenins and triterpene saponins [19,20]. Saponins are plant glycosides that were found to induce cell cycle arrest in a human breast cancer cell line and initiation of apoptosis in a leukemia cell line [21]. Additionally, certain classes of saponins can sequester serum cholesterol and modulate the immune response [22]. The individual properties of Ag and AA were considered for the synthesis of a novel AgNP using aqueous leaf extracts of the plant.

The A549 cell line (human lung carcinoma) is well characterized and extensively used in *in vitro* nanotoxocity studies [23]. A recent study postulated that the induction of ROS and alterations in mt membrane permeability were possible mechanisms by which AgNP exerted its toxic effects in A549 cells [24]. The aim of this study was to investigate the effects of AA_AgNP_ on lung cancer cells. It was hypothesized that AA_AgNP_ induced cell death by apoptosis as a result of AA_AgNP_ mediated generation of ROS. We report on a possible mechanism by which AA_AgNP_ induced apoptosis in the A549 cells.

## Results

### Cell viability assay

**Figure 1:**
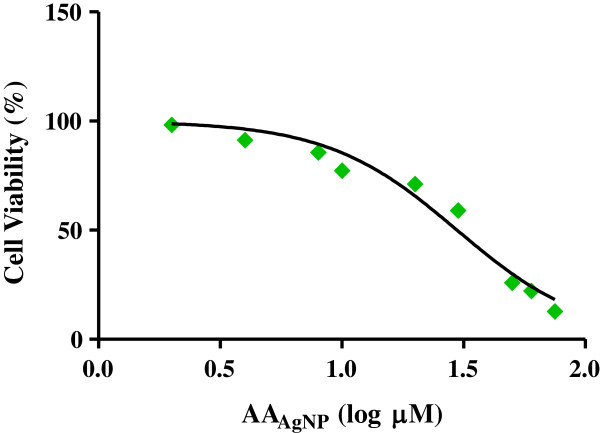
**A dose-dependent decline in A549 cell viability after AA**_**AgNP **_**treatment.** The MTT assay was used to determine effect of AA_AgNP_ on A549 cell viability. Cells were exposed to AA_AgNP_ in the range 0-75 μg/ml for 6h, after which the formazan product was quantified spectrophotometrically. A distinct dose dependent effect was observed where AA_AgNP_ at lower concentrations induced minimal cell death. A gradual decrease in cell viability occurred with increasing AA_AgNP_ concentration (95% Cl = 31.96 to 57.72). An IC_50_ value of 43 μg/ml was obtained and used for subsequent assays.

Toxicity of AA_AgNP_ to A549 cells was determined using the MTT assay. A dose-dependent decline in cell viability was observed using AA_AgNP_ concentrations in the range 0 to 75 μg/ml for 6h (95% Cl = 31.96 to 57.72) (Figure 1). An IC_50_ value of 43 μg/ml was obtained and used in all subsequent assays.

### ATP analysis

**Figure 2:**
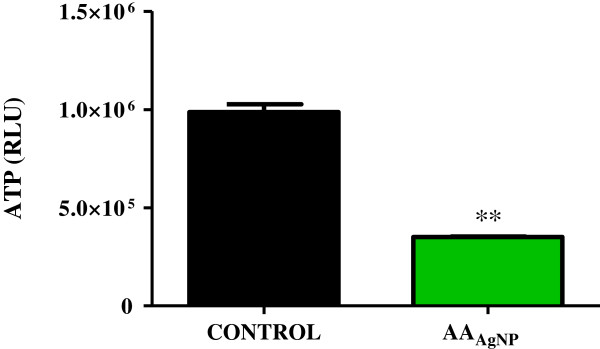
**Levels of ATP in control and AA**_**AgNP **_**treated A549 cells.** Cellular ATP levels were detected by the ATP CellTitre Glo assay (Promega, Madison, USA). A significant decline in ATP was noted after treatment with AA_AgNP_ (350000 ± 1500RLU; 95% CL = 330000 to 370000) compared to the control cells (990000 ± 40000RLU; 95% Cl = 480000 to 1500000; *p* = 0.0040). RLU: relative light units.

Levels of ATP were assessed using luminometric assay. Silver nanoparticles of AA significantly reduced ATP levels with a 2.5-fold decrease (350,000 ± 1500RLU; 95% CL = 330,000 to 370,000) compared to the control (990,000 ± 40,000RLU; 95% Cl = 480,000 to 1,500,000; *p* = 0.0040) (Figure 2).

### Oxidative status

**Figure 3:**
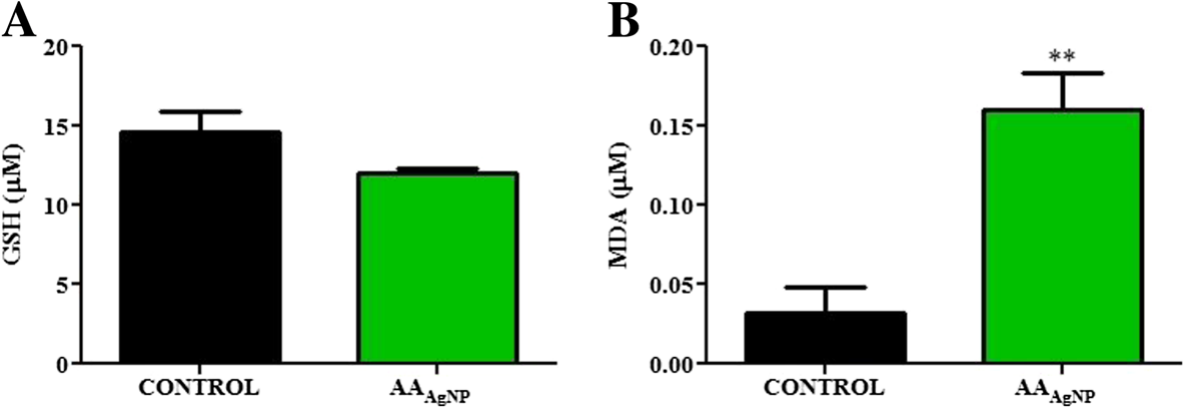
**A) Levels of GSH and B) MDA (lipid peroxidation) in AA**_**AgNP **_**treated and untreated cells.** The oxidative status of A549 cells were assessed by quantifying GSH and MDA. Levels of the anti-oxidant GSH were measured by the GSH-Glo™ Glutatione assay (Promega, Madison, USA). AA_AgNP_ caused a decrease in GSH (12 ± 0.24 μM) compared to untreated cells (15 ± 1.3 μM, 95% Cl = −1.0 to 6.2; *p* = 0.1184). The TBARS assay was utilized to quantify MDA levels as marker for lipid peroxidation induced by ROS. A significant elevation in MDA levels were found in cells exposed to AA_AgNP_ (0.16 ± 0.023 μM) compared to the control (0.032 ± 0.016 μM, 95% Cl = −0.21 to −0.049; *p* = 0.0098).

Glutathione concentrations were measured as a marker for intracellular anti-oxidant capacity. The concentration of GSH was higher in untreated cells (15 ± 1.3 μM) compared to AA_AgNP_ treated cells (12 ± 0.24 μM, 95% Cl = −1.0 to 6.2; *p* = 0.1184) (Figure 3A). Lipid peroxidation (MDA) was significantly 5-fold higher in cells exposed to AA_AgNP_ (0.16 ± 0.023 μM) compared to controls (0.032 ± 0.016 μM, 95% Cl = −0.21 to −0.049; *p* = 0.0098) (Figure 3B).

### Analysis of caspases

AA_AgNP_ significantly increased the activities of caspase-3/-7 (1.7-fold, 95% Cl = −1,000,000 to −260,000; *p* = 0.0180) and −9 (1.4-fold, 95% Cl = −610,000 to −220,000; *p* = 0.0117) compared to the control. The activity of caspase-8 however was significantly decreased by AA_AgNP_ compared to untreated cells (2.5-fold, 95% Cl = 550,000 to 840,000; *p* = 0.0024) (Table 1).

**Table 1:** **Caspase activity in AA**_**AgNP**_**treated cells**

	**Control**	**AA**_**AgNP**_	
	**Mean RLU ± sem**	**Mean RLU ± sem**	***p*value**
**CASPASE-8**	1.2 × 10^6^ ± 2.6 × 10^4^	4.6 × 10^5^ ± 2.3 × 10^4^	0.0024**
**CASPASE-9**	9.2x10^5^ ± 3.3 × 10^4^	1.3x10^6^ ± 3.1 × 10^4^	0.0117*
**CASPASE-3/-7**	9.2x10^5^ ± 4 × 10^3^	1.6x10^6^ ± 8.6 × 10^4^	0.0180*

RLU: relative light units.

### Flow cytometry

**Figure 4:**
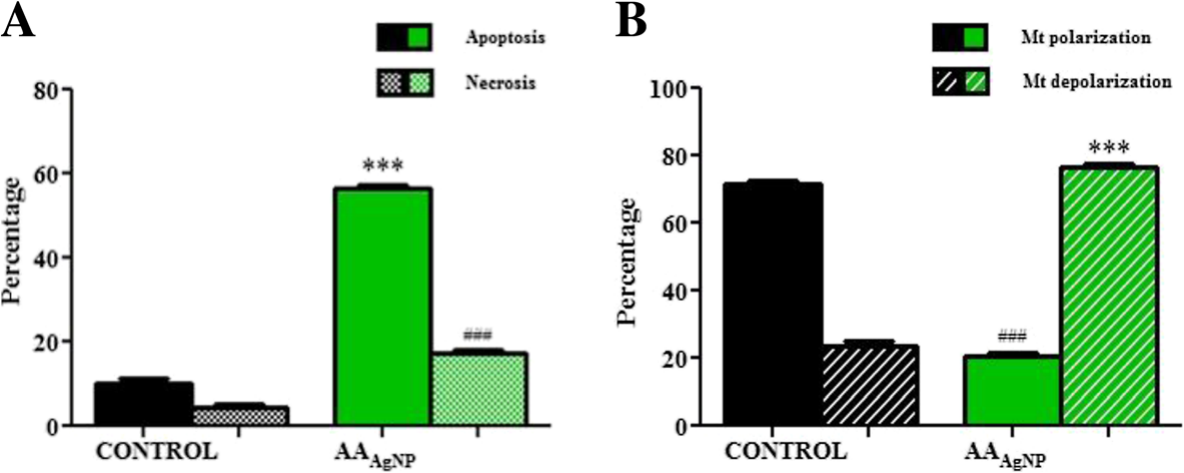
**A) Percentages of apoptotic and necrotic cells and B) Mt depolarization after treatment with AA**_**AgNP**_**.** Flow cytometry was used to examine PS translocation (annexin-V-Fluos assay (Roche)) and mt membrane integrity (BD™ MitoScreen kit (BD Biosciences). A significant increase in apoptosis (57 ± 0.59% vs. control: 10 ± 0.84%, 95% Cl = −50 to −43; *p* < 0.0001) and necrosis (17 ± 0.79% vs. control: 4.6 ± 0.70%, 95% Cl = −16 to −9.2; *p* < 0.0001) following treatment was seen. Furthermore, mt membrane depolarization was significantly higher in AA_AgNP_ treated cells (77 ± 0.88% vs. control: 23 ± 1.8%, 95% Cl = 44 to 57; *p* < 0.0001).

Silver nanoparticles of AA significantly increased PS translocation A549 cell compared to the control (57 ± 0.59% vs. 10 ± 0.84%, 95% Cl = −50 to −43; *p* < 0.0001) (Figure 4A). Additionally, the percentage of necrotic cells were also significantly higher in AA_AgNP_ treated cells (17 ± 0.79% vs. control: 4.6 ± 0.70%, 95% Cl = −16 to −9.2; *p* < 0.0001) (Figure 4A). The apoptosis inducing potential of AA_AgNP_ was further verified in disrupting mt ∆ Ψ. The treated cells had significantly higher mt depolarization compared to untreated cells (77 ± 0.88% vs. 23 ± 1.8%, 95% Cl = 44 to 57; *p* < 0.0001) (Figure 4B).

The expression of CD95 was significantly down regulated (2-fold; 2.8 ± 0.58% vs. control: 5 ± 0.44%, 95% Cl = 0.13 to 4.2; *p* = 0.0416) (Table 2). In contrast, AA_AgNP_ significantly increased the expression of smac/DIABLO, a pro-apoptotic protein (29 ± 0.32%) compared to the control (18 ± 0.66%, 95% Cl = −13 to −8.5; *p* < 0.0001) (Table 2). Additional files contain the histograms for extra- and intracellular staining (see Additional file 1: Figure S1 and Additional file 2: Figure S2 respectively).

**Table 2:** **Surface expression of CD95 and intracellular smac/DIABLO in A549 cells treated with AA**_**AgNP**_**as determined flow cytometrically**

	**Control**	**AA**_**AgNP**_	
	**Mean % ± sem**	**Mean % ± sem**	***p*value**
**FITC + ve (CD95)**	5 ± 0.44	2.8 ± 0.58	0.0416*
**APC + ve (smac/DIABLO)**	18 ± 0.66	29 ± 0.32	<0.0001***

### Comet assay

**Figure 5:**
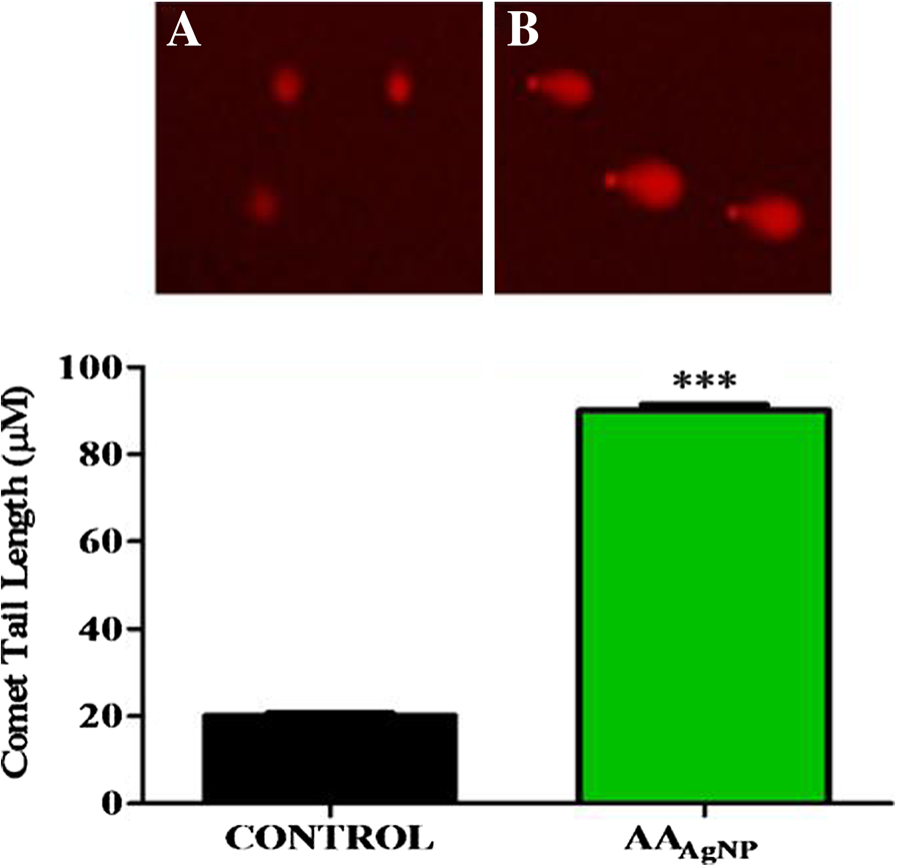
**Fragmentation of DNA in cells exposed to AA**_**AgNP **_**(B) and untreated control cells (A) (100x).** To assess the genotoxic effects of AA_AgNP_, the comet assay was used. Fragmentation of DNA (indicated by comet tails) was significantly induced by AA_AgNP_ treatment (90 ± 1.3 μm) compared to control cells (20 ± 0.71 μm, 95% Cl = −73 to −67; *p* < 0.0001).

Silver nanoparticles of AA were significantly genotoxic to A549 cells as noted by the increased DNA fragmentation. Comet tail lengths were significantly longer in treated (90 ± 1.3 μm) compared to untreated cells (20 ± 0.71 μm, 95% Cl = −73 to −67; *p* < 0.0001) (Figure 5).

### Western blot analysis

**Figure 6:**
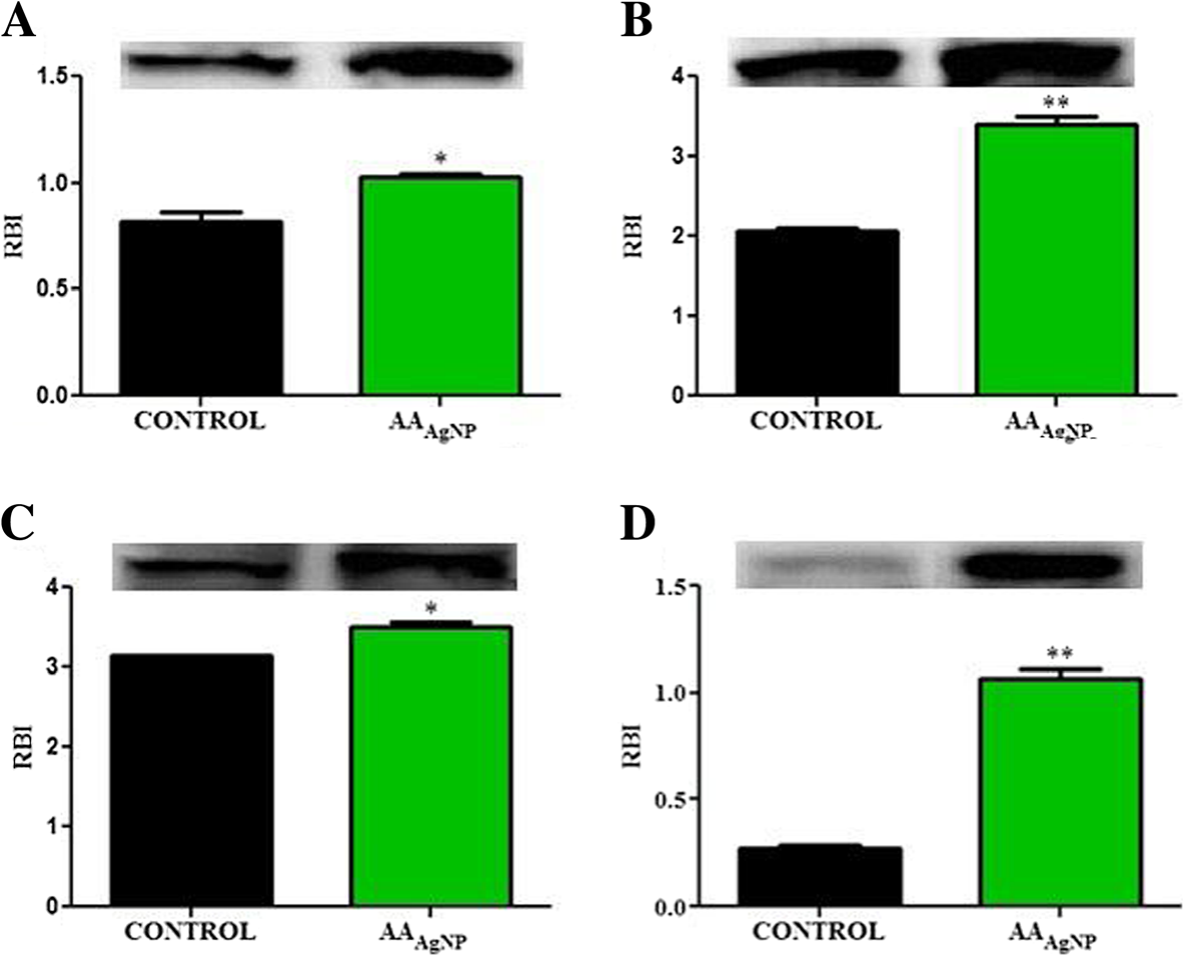
**The expression of apoptotic proteins (A) p53, B) PARP-1, C) bax and D) smac/DIABLO) in AA**_**AgNP **_**treated and untreated cells.** Western blotting assessed the expression of selected apoptotic proteins. Levels of p53 were significantly higher post AA_AgNP_ exposure (1.0 ± 0.018RBI vs. control: 0.82 ± 0.045RBI, 95% Cl = −0.42 to −0.00037; *p* = 0.0498). Cleavage of PARP-1 was distinct with significant increase in the 24kDa fragment after AA_AgNP_ treatment (3.5 ± 0.069RBI vs. control: 3.1 ± 0.0043RBI, 95% Cl = −0.65 to −0.058; *p* = 0.0359). The expression of bax was significantly higher in cells that were treated with AA_AgNP_ (3.4 ± 0.11RBI) compared to untreated cells (2.1 ± 0.047RBI, 95% Cl = −1.9 to −0.81; *p* = 0.0083). AA_AgNP_ treated cells presented with a 4.1-fold greater band intensity for smac/DIABLO (1.1 ± 0.045RBI vs. control: 0.27 ± 0.010RBI, 95% Cl = −0.99 to −0.59; *p* = 0.0033). Protein bands were standardized against β-actin.

The expression of selected apoptotic proteins and validation of flow cytometric intracellular stained smac/DIABLO was determined by western blotting. The expression of p53 was observed to be significantly higher post AA_AgNP_ exposure (1.0 ± 0.018RBI vs. control: 0.82 ± 0.045RBI, 95% Cl = −0.42 to −0.00037; *p* = 0.0498). A significant increase in the 24kDa fragment of PARP-1 was noted after AA_AgNP_ treatment (3.5 ± 0.069RBI vs. control: 3.1 ± 0.0043RBI, 95% Cl = −0.65 to −0.058; *p* = 0.0359). Evaluation of bax showed higher levels of the protein in cells that were treated with AA_AgNP_ (3.4 ± 0.11RBI) compared to untreated cells (2.1 ± 0.047RBI, 95% Cl = −1.9 to −0.81; *p* = 0.0083). Highly significant differences were seen in the expression of smac/DIABLO between experimental and control cells. Silver nanoparticles of AA treated cells presented with a 4.1-fold greater band intensity (1.1 ± 0.045RBI vs. control: 0.27 ± 0.010RBI, 95% Cl = −0.99 to −0.59; *p* = 0.0033) (Figure 6).

## Discussion

Several pathological syndromes such as liver failure, stroke or heart attack are associated with abrupt death of tissue or organs as a result of apoptotic dysregulation. Conversely, the survival of abnormal cells, due to aberrant apoptosis, may lead to tumorigenesis [25]. Apoptosis is commonly altered in cancerous cells, which have the ability to evade the apoptotic cascade.

Silver nanoparticles of AA significantly increased PS externalization, a transmembrane glycoprotein, in the treated A549 cells (Figure 4A).

The signal transduction of apoptosis involves a cascade of initiator and executioner caspases [26]. Executioner caspases-3/-7 cleave specific substrates leading to alteration changes linked with apoptosis and ultimately cell death [27]. Initiator caspses-8 and −9 are responsible for the activation of executioner caspases. Silver nanoparticles of AA significantly up regulated the activities of caspases-3/-7 and −9 (Table 1). Furthermore AA_AgNP_ increased DNA fragmentation (Figure 5)-an end stage characteristic of apoptosis. In response to this DNA damage, the nuclear enzyme PARP-1 catalyzes the transfer of NAD^+^ to a specific set of nuclear substrates [28]. During apoptosis, PARP-1 is cleaved, by executioner caspases-3/-7, to a 24kDa DNA binding domain and an 89kDa fragment containing catalytic activity. Silver nanoparticles of AA was responsible for the cleavage of PARP-1 as evidenced by the significantly increased expression of 24kDa fragment compared to untreated control cells (Figure 6B).

Mitochondria play an important role in apoptosis, via the intrinsic apoptotic program. An initial step for activation of the intrinsic apoptotic pathway is the depolarization of the mt membrane. Depolarized mt is as a result of the formation of mt permeability transition (PT) pores [29]. Mitochondrial PT has been associated with various metabolic consequences such as halted functioning of the electron transport chain with associated elevation in ROS and decreased production of ATP [30]. Bax, a pro-apoptotic protein of the Bcl-2 family, translocates from the cytosol to the outer mt membrane during apoptosis where it interacts with lipids and induces mt PT pores. A significant increase in mt depolarization was observed after AA_AgNP_ treatment (Figure 4B), with an accompanying decrease in ATP concentration (Figure 2). The high levels of bax expression (Figure 6C), high mt depolarization and decreased ATP suggest that AA_AgNP_ induces cellular apoptosis in cancerous lung cells via the intrinsic apoptotic pathway.

Silver has a high affinity for thiol (−SH) groups [31]. In this study, the levels of cysteine-rich GSH were decreased whilst lipid peroxidation was significantly elevated by AA_AgNP_ (Figure 3). This oxidant/anti-oxidant imbalance has previously been documented as an apoptotic mechanism by AgNP mediated cytotoxicity [32,33].

The extrinsic apoptotic pathway is mediated by CD95 death receptor, which recruits Fas-associated protein with death domain (FADD) adapter protein. The adapter protein FADD binds to and activates caspase-8 via the formation of a death-inducing signaling complex [25]. The results of this study show that CD95 expression (Table 2) and caspase-8 activity (Table 1) was significantly decreased by AA_AgNP_. The extracts of AA are rich in saponins, which will promote rapid entry of the AA_AgNP_ into the cells resulting in mt mediated intrinsic apoptotic pathway. A well characterized biological action of saponins is their ability to induce cell membrane permeabilization [34]. Decreased ATP concentrations and increased MDA as a result of ROS may be due to disruptions in the mt respiratory chain. Nanoparticles preferentially localize in mt and cause oxidative stress as well as potentiate structural damage [35,36]. Various studies have associated AgNP toxicity with mt damage [37,38].

Several pro-apoptotic molecules are released from the mt during apoptosis. In the presence of ATP, mt released cytochrome c associates with apoptotic protease-activating factor (Apaf)-1 in the cytosol inducing its oligomerization. An apoptosome is then formed with the oligomeric Apaf-1 complex and procaspase-9, inducing the activation of caspase-9, which in turn activates effector caspases-3 and −7 [26]. An interesting finding in this study was that although ATP levels were reduced post AA_AgNP_ treatment, the activity of caspase-9 was still elevated (Table 1).

A class of molecules involved in the regulation of apoptosis is the inhibitor of apoptosis (IAP) proteins. These proteins avert cell death by suppressing the activity of caspases. X-chromosome-linked inhibitor of apoptosis (XIAP) is the most well characterized member of IAPs [25]. The ability of IAPs to act as endogenous suppressors of procaspase activation is attributed to the presence of domains referred to as baculoviral IAP repeats (BIR). In particular, BIR3 and a region adjacent to BIR2 are responsible for the inhibition of caspases-9, and −3 and −7 respectively. Smac/DIABLO, a mt protein, is able to abolish the inhibitory effects of XIAP [26]. Both smac/DIABLO and caspases-3,-7 and −9 contain IAP-binding motifs that fit into the BIR domains of XIAP. Thus, smac/DIABLO is able to relieve inhibition by replacing and releasing caspases-3, -7 and −9 from the XIAP inhibitory complex [25,26]. We postulated that AA_AgNP_ released smac/DIABLO from the mt. The increased intracellular staining (Table 2) and expression of smac/DIABLO by western blotting (Figure 6D) confirmed that AA_AgNP_ induced its release from the mt.

The p53 protein mediates a range of anti-proliferative processes in response to different stress stimuli by directly activating apoptosis and promoting the release of bax [39] and inducing executioner caspase activity [40] Also, p53 interferes with mt integrity and function leading to the release of pro-apoptotic molecules and the generation of ROS [41]. This study clearly shows the increased expression of p53 after addition of AA_AgNP_ to cells (Figure 6A).

## Conclusion

In conclusion, this novel AA_AgNP_ possesses potent pro-apoptotic potential. We have shown, mechanistically, that AA_AgNP_ activates the intrinsic apoptotic pathway in A549 lung carcinoma cells. The findings of this study suggest the potential for AA_AgNP_ in drug development against cancer. None the less, further studies need to be conducted to ascertain if the effects of AA_AgNP_ are consistent in other cancerous cell lines and also non-toxic to healthy systems.

## Materials and methods

### Materials

A549 cells were purchased from Highveld Biologicals (Johannesburg, South Africa). Cell culture reagents were purchased from Whitehead Scientific (Johannesburg, South Africa). LumiGLO® chemiluminescent substrate kit was purchased from Gaithersburg (USA) and western blot reagents were purchased from Bio-Rad (USA). All other reagents were purchased from Merck (South Africa).

### Synthesis of AA_AgNP_

Synthesis and characterization of AA_AgNP_, described by Gengan *et al*. 2013, was conducted at the Steve Biko campus, Durban University of Technology, Durban, South Africa. A one-pot green synthesis technique was used [42]. Briefly, fresh leaves of AA were extracted with deionized water. The crude extract was filtered and the supernatant was allowed to react with silver nitrate solution at room temperature (RT). Silver nanoparticles of AA solution (pH 7) were then characterized using UV spectrometry. Particle size was determined by transmission electron microscopy. To assess the interaction between nanosilver and compounds of the aqueous extracts of AA leaves, Fourier transform infrared spectrophotometry was employed [42]. Ethical approval from the Biomedical Research Ethics Administration Office of the University of KwaZulu-Natal (Reference number: BE050/08) was obtained.

### Cell culture and exposure protocol

The A549 cells were cultured in Eagle’s minimum essential medium supplemented with 1% L-glutamine, 1% penstrepfungizone and 10% fetal bovine serum. Cultures were maintained at 37°C with 5% CO_2_. For the 3-(4,5-Dimethyl-2-thiazolyl)-2,5-diphenyl-2H-tetrazolium bromide (MTT) assay, cells were seeded into a 96-well microtitre plate, allowed to attach overnight and treated with AA_AgNP_ solution (0-75 μg/ml). For flow cytometry assays, caspase, ATP and lipid peroxidation assays, cells were cultured to 90% confluency in 25 cm^2^ tissue flasks and treated with AA_AgNP_. For the GSH assay, cells were plated in 96-well microtitre plates and allowed to attach overnight, followed by treatment with AA_AgNP_. For western blot analysis and the comet assay, cells were grown to 90% confluency in 6-well culture plates and treated with AA_AgNP_.

### Cell viability assay

Cell viability was determined using the MTT assay. Approximately 20,000 cells (in six replicates) were used for exposure to AA_AgNP_ concentrations in the treatment range. After incubation with AA_AgNP_ for 6 h, cells were washed twice with 0.1M phosphate buffer saline (PBS) and incubated with MTT salt solution (5 mg/ml in 0.1M PBS) and complete culture medium (37°C, 4 h). Thereafter, 100 μl of dimethyl sulfoxide was added to each well and incubated (37°C, 1 h). Optical density of the formazan product was measured using a spectrophotometer (Bio-tek μQuant) at 570/690 nm. The results were expressed as percentage cell viability vs. concentration of AA_AgNP_, from which the half maximal inhibitory concentration (IC_50_) was determined.

### ATP assay

Cells (20,000/well in six replicates) were aliquoted in an opaque 96-well microtitre plate to which the ATP CellTitre Glo (Promega, Madison, USA) reagent (50 μl) was added and allowed to react in the dark (30 min, RT). After incubation, the luminescent signal proportional to the cellular ATP content was detected with a Modulus™ microplate reader (Turner Biosystems, Sunnyvale, USA). The results were expressed as mean relative light units (RLU).

### Glutathione assay

The GSH-Glo™ Glutatione assay (Promega, Madison, USA) was utilized to quantify intracellular GSH levels. Subsequent to treatment of cells (10,000 cells/well in six replicates) in an opaque 96-well microtitre plate, culture medium was removed and 25 μl of 1X GSH-Glo™ reagent (prepared according to manufacturer’s guidelines) was added to each well. Glutathione standards (0-5M) were serially diluted (two-fold) from a 5 mM stock in deionized water. After brief mixing on a shaker and 30 min incubation at RT, 100 μl of Luciferin detection reagent was added to the wells (15 min, RT). Luminescence was detected on a Modulus™ microplate luminometer (Turner Biosystems, Sunnyvale, USA). A calibration curve was constructed and sample GSH concentrations (μM) were calculated.

### Lipid peroxidation

To investigate the AA_AgNP_-mediated generation of reactive oxygen species (ROS), malondialdehyde (MDA-a product of lipid peroxidation) levels were measured using the thiobarbituric acid reactive substances (TBARS) assay. Briefly, the following was added to a set of test tubes: 200 μl of 2% H_3_PO_4_, 400 μl of 7% H_3_PO_4_, 400 μl of TBA/BHT solution and 200 μl of 1M HCL. Following treatment of cells (50,000 cells/well) in a 6-well plate, supernatants were recovered. For test samples, 100 μl of cell supernatant (in triplicate) was then added to each test tube. A positive control was prepared by adding 1 μl of MDA to a test tube. All tubes were incubated in a water bath (100°C, 15 min) and after cooling; butanol (1.5 ml) was added to each tube, vortexed for 10 seconds and allowed to separate into two distinct phases. Approximately 800 μl of the upper butanol phase was then transferred to 1.5 ml tubes and centrifuged (840 x g, 24°C; 6 min). To a 96-well micotitre plate, 100 μl of supernatant was transferred in six replicates and the absorbance read using a spectrophotometer (Bio-tek μQuant) at 532/600 nm. The mean absorbance was divided by the extinction co-efficient (156 mM^-1^) and results were expressed as μM concentrations.

### Assessment of caspase activity

Caspase-3/-7, -8 and -9 activities were detected with Caspase-Glo® assays (Promega, Madison, USA). As per manufacturer’s protocol, Caspase-Glo®-3/-7, -8 and -9 reagents were reconstituted and added to wells (in six replicates) of an opaque 96-well microtitre plate (40 μl of reagent per 100 μl of 10,000 cells/well). Samples were mixed and incubated in the dark (30 min, RT). The luminescent signal was measured on a Modulus™ microplate luminometer (Turner Biosystems, Sunnyvale, USA). Caspase-3/-7, -8 and -9 activities were expressed as relative light units (RLU).

### Annexin-V-FLUOS assay

The annexin-V-Fluos assay (Roche) was used to determine phosphatidylserine (PS) translocation. To each flow cytometry tube, 100 μl of staining buffer, 100 μl of annexin-V-Fluos labeling solution (annexin-V: propidium iodide (PI): staining buffer (1:1:50 vol/vol/vol)) and 100 μl of cell suspension was added, and incubated in the dark (15 min, RT). Samples were analyzed on a FACS Calibur (BD Biosciences) flow cytometer. Data were analyzed using CellQuest PRO v4.02 software (BD Biosciences). Cells were gated to exclude cellular debris using FlowJo v7.1 software (Tree Star, Inc). Approximately 50,000 events were analyzed for apoptotic (annexin-V + ve, PI -ve), necrotic (annexin-V + ve, PI + ve) and live cells (annexin-V -ve, PI -ve). The results were expressed as percentage of the total events.

### JC-1 MitoScreen assay

Mitochondrial membrane potential (∆ Ψ) was assayed with the BD™ MitoScreen kit (BD Biosciences). JC-1, a cationic dye, is sensitive to ∆ Ψ and accumulates in mt with polarized membranes. JC-1 working solution was prepared and 100 μl added to each flow cytometry tube, followed by 100 μl of cell suspension. Tubes were incubated at 37°C with 5% CO_2_ for 15 min, after which 100 μl of JC-1 wash buffer was added. Approximately 50,000 events were analyzed for mt depolarization. A FACS Calibur flow cytometer was used and data were analyzed using CellQuest PRO v4.02 software. Cells were gated to exclude cellular debris using FlowJo v7.1 software. The results were expressed as percentage of the total events.

### Comet assay

The comet assay was used to determine DNA fragmentation in the AA_AgNP_ treated lung cells. Briefly, three slides per sample were prepared with a first layer containing 400 μl of 1% low melting point agarose (LMPA, 37°C), a second layer of 25 μl of cells from each sample with 175 μl 0.5% LMPA (37°C) and a third layer containing 200 μl of 1% LMPA (37°C). Cover slips were removed and slides were subjected to lysis (4°C, 1 hr, protected from light) by being submerged in cells lysis buffer (2.5M NaCl, 100 mM EDTA, 1% Triton X-100, 10 mM Tris (pH 10) and 10% DMSO). The slides were equilibrated in electrophoresis buffer (300 mM NaOH, 1 mM Na_2_EDTA, pH 13; 20 min) then electrophoresced (300 mA, 25V, 35 min) after which slides were washed three times (0.4M Tris, pH 7.4; 5 min) and finally stained with 40 μl ethidium bromide. Cover slips were placed onto slides and maintained overnight at 4°C. Slides were viewed using a fluorescent microscope (Olympus IXSI inverted microscope/510-560 nm excitation and 590 nm emission wavelengths). Images were captured and comet tails of 50 cells were measured using Life Science-Soft Imaging System (analySIS® v5). The results were expressed as mean tail length in μm.

### CD95 (Fas) analysis

CD95 receptor expression was determined by cell surface staining on a flow cytometer. Briefly, 100 μl of 0.1M PBS and 1 μl of fluorescein isothiocyanate (FITC) conjugated mouse anti-human CD95 antibody (BD Pharmingen, 555673) was added to 100 μl of cell suspension. Reaction mixture was incubated in the dark for 15 min at RT, thereafter run on a FACS Calibur. Data were analyzed using CellQuest PRO v4.02 software. Cells were gated to exclude cellular debris using FlowJo v7.1 software. The results were expressed as a percentage.

### Intracellular staining

Intracellular levels of smac/DIABLO were assessed by flow cytometry. Cells were rendered permeable by incubation in Fix and Perm medium A (Caltag) (15 min in the dark), after which they were treated with Fix and Perm medium B (Caltag) containing monoclonal anti-smac/DIABLO primary antibody (smac/DIABLO, ab110291) (1:500; 30 min, RT). After washing, cells were re-suspended in medium B containing allophycocyanin (APC) conjugated anti-mouse secondary antibody (Thermo Scientific, 31430) (1:100; 15 min, RT) protected from light. Cells were then washed, re-suspended in 0.1M PBS and run on a FACS Calibur. Data were analyzed using CellQuest PRO v4.02 software. Cells were gated to exclude cellular debris using FlowJo v7.1 software. The results were expressed as percentage.

### Western blot analysis

Cytobuster™ reagent (Novagen) supplemented with protease and phosphatase inhibitors (Roche, 05892791001 and 04906837001 respectively) was used for protein isolation. Cytobuster (200 μl) was added to the cells (4°C, 10 min) and centrifuged (180 x g; 4°C, 10 min) to obtain a crude protein extract. Protein samples were quantified using the bicinchoninic assay and standardized to 1 mg/ml. Samples (25 μl) were electrophoresced on 7.5% sodium dodecyl sulfide-polyacrylamide gel electrophoresis gels and thereafter transferred to nitrocellulose membranes. Membranes were blocked with 3% bovine serum albumin (BSA) in Tris buffer saline (20 mM Tris–HCl (pH 7.4), 500 mM NaCl and 0,01% Tween 20 (TBST)) for 1 h, and incubated with primary antibody (p53, ab26; bax, ab5714; parp-1, ab110915; smac/DIABLO, ab110291 and β-actin, ab8226; 1:1,000) in 1% BSA in TBST at 4°C overnight. Membranes were then washed thrice (10 ml TBST, 15 min) and treated with horseradish peroxidase-conjugated secondary antibody (mouse, ab97046; 1:2,000) (1 h, RT). Membranes were washed again 3 times (TBST, 15 min) and immunoreactivity was detected by the LumiGLO® chemiluminescent substrate system (KPL) with the Uvitech Image Documentation System (UViTech Alliance 2.7). Protein bands were analyzed with the UViBand Advanced Image Analysis software (UViTech v12.14). The results were expressed as mean relative band intensity (RBI).

### Statistical evaluation

Statistical analyses were performed using GraphPad Prism version 5.00 software package (GraphPad PRISM®). Data are expressed as mean ± standard error of the mean (sem). Comparisons were made using unpaired t tests. Statistical significance was set at 0.05.

## Competing interests

The authors declare that they have no competing interests.

## Authors’ contributions

The design of the study was proposed by AAC. RMG and KA were responsible for the synthesis and characterization of AA_AgNP_. RG and AP carried out cell culture, subsequent experimental procedures as well as statistical analyses. The manuscript was drafted by RG, AP and AAC. All authors read and approved the final manuscript.

## Supplementary Material

Additional file 1: Figure S1 Extracellular staining-flow cytometry was used to evaluate the expression of CD95 (Fas receptor). Three replicates were done for both treated (B1-B3) and untreated (A1-A3) cells. AAAgNP significantly down regulated the expression of CD95 in A549 cells compared to the control (2.8 ± 0.58% vs. 5 ± 0.44%; *p* = 0.0416; 95% Cl = 0.13 to 4.2). Click here for file

Additional file 2: Figure S2 Levels of smac/DIABLO, a pro-apoptotic protein, were determined flow cytometrically using an intracellular staining assay. A significantly higher expression of smac/DIABLO was observed in A549 cells after treatment with AAAgNP (B1-B3) compared to untreated cells (A1-A3) (29 ± 0.32% vs. 18 ± 0.66%; *p* < 0.0001; 95% Cl = -13 to -8.5). Click here for file
