# Supplementary figures and images for: Silver nanoparticles of Albizia adianthifolia: the induction of apoptosis in human lung carcinoma cell line
Source: J Nanobiotechnology. 2013 Feb 18;11:5. doi: 10.1186/1477-3155-11-5 (PMC3606379; doi:10.1186/1477-3155-11-5)

## Extracellular staining

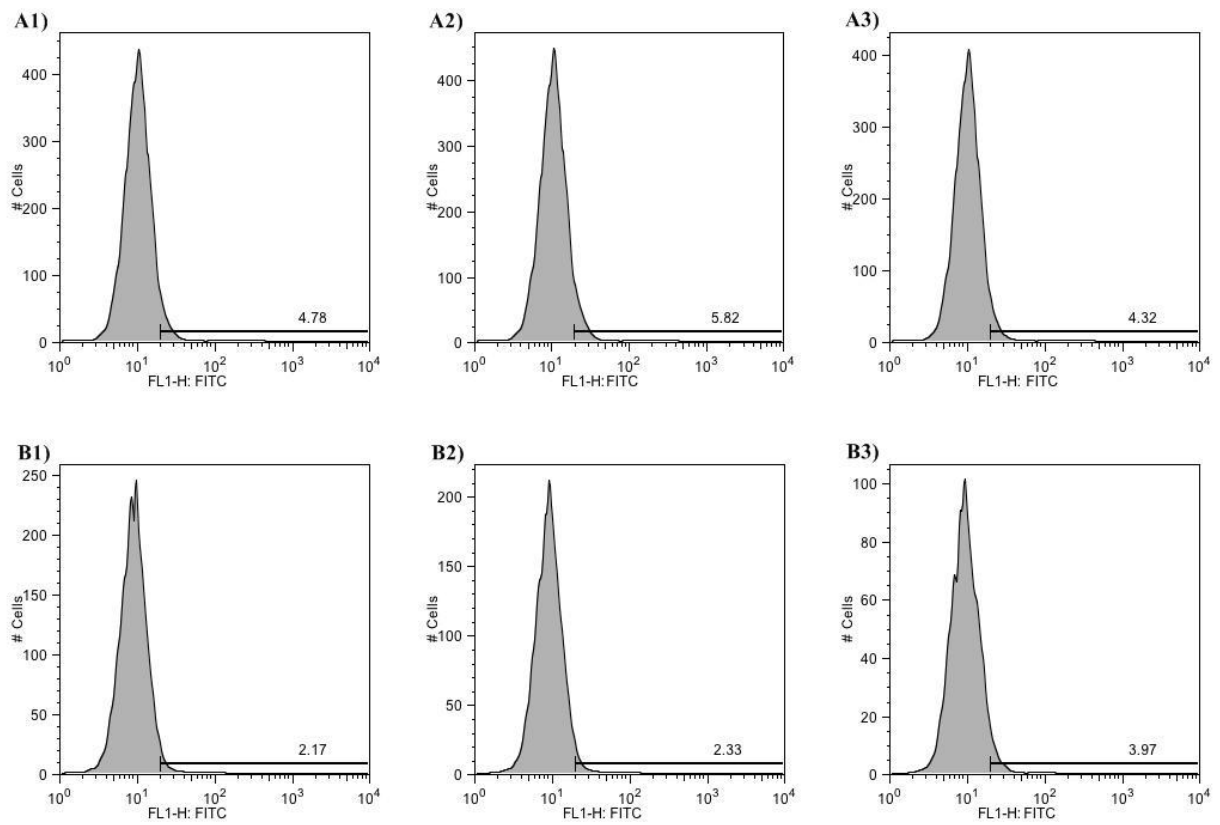

Supplement: Additional file 1: Figure S1 — Extracellular staining-flow cytometry was used to evaluate the expression of CD95 (Fas receptor). Three replicates were done for both treated (B1-B3) and untreated (A1-A3) cells. AAAgNP significantly down regulated the expression of CD95 in A549 cells compared to the control (2.8 ± 0.58% vs. 5 ± 0.44%; p = 0.0416; 95% Cl = 0.13 to 4.2). [file 1477-3155-11-5-S1.pdf]

## Intracellular staining

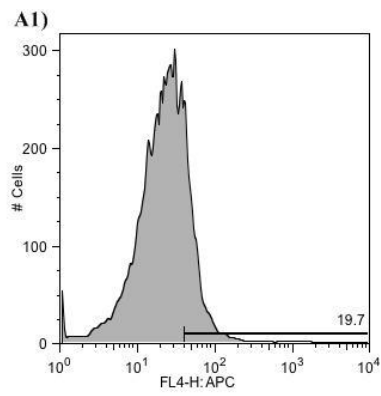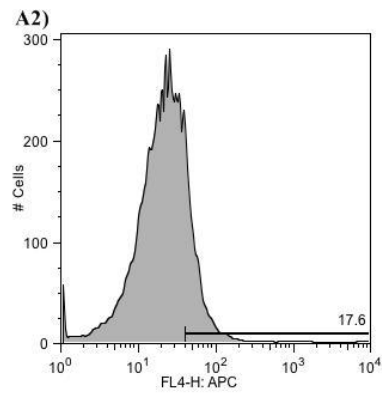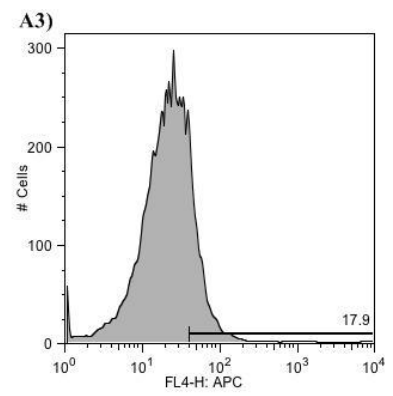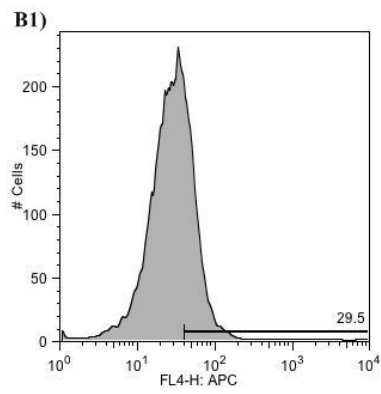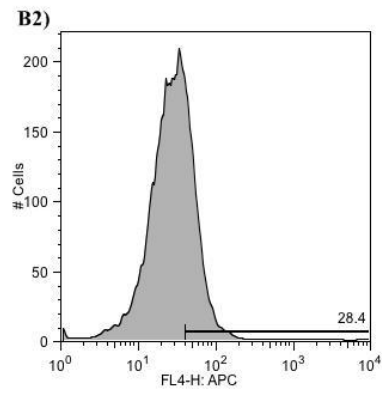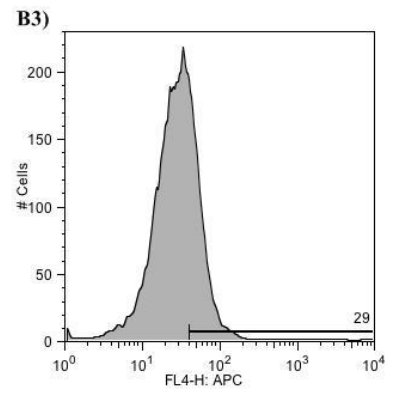

Supplement: Additional file 2: Figure S2 — Levels of smac/DIABLO, a pro-apoptotic protein, were determined flow cytometrically using an intracellular staining assay. A significantly higher expression of smac/DIABLO was observed in A549 cells after treatment with AAAgNP (B1-B3) compared to untreated cells (A1-A3) (29 ± 0.32% vs. 18 ± 0.66%; p < 0.0001; 95% Cl = -13 to -8.5). [file 1477-3155-11-5-S2.pdf]
